# Organ Donation in the Emergency Department: Awareness and Opportunities

**DOI:** 10.7759/cureus.49746

**Published:** 2023-11-30

**Authors:** Yahia Y Akeely, Mojahid M Al Otaibi, Saleh A Alesa, Nader N Bokhari, Tariq A Alghamdi, Muneera S Alahmari, Nada K AlRasheed

**Affiliations:** 1 Emergency Department, Security Forces Hospital, Riyadh, SAU

**Keywords:** organ failure, emergency department, organ transplantation, organ donation, emergency physician

## Abstract

Background

The only cure for end-organ failure is transplantation. Unfortunately, there are fewer organ donors than patients. Currently, the majority of organ donations come from live or brain-dead donors. In order to expand the pool of potential organ donors, the emergency department should be utilized effectively.

Objectives

The primary goal of this research is to determine emergency physicians' knowledge, awareness, and attitude about organ donation.

Methodology

A cross-sectional study was conducted through different hospitals in Saudi Arabia. It includes 106 physicians in the adult emergency departments.

Results

The majority (84.9%) of the participants never reported any case in the emergency department as a potential case for organ donation. In addition, 54.8% of the participants report having little to no knowledge of the ethical issues of organ donation. Furthermore, 66.1% of respondents claim to have little to no knowledge of the goals and duties of the SCOT (Saudi Center for Organ Transplantation). It was interesting to see that 96.2% of the participants reported that their organizations do not have any policies or procedures in place regarding organ donations. Regarding education, 99 (93.4%) physicians did not participate in any organ donation course, training, or teaching program about organ donation. At the same time, 67 (63.2%) physicians concurred that participation in a training program is essential. Moreover, 68 (64.2%) physicians believed that organ donation should be a part of every end-of-life case. In order to improve the donation process in the emergency department, 88 (83%) physicians would want a well-established program with defined policies and procedures.

Conclusion

According to our findings, the emergency physician has inadequate expertise and information on organ donation rules and procedures, which has resulted in a missed opportunity to recruit more potential donors. We recommend instituting clear policy and procedures and educating the physicians and all emergency medicine staff to have better outcome.

## Introduction

Organ transplantation is a definite and life-saving medical procedure for those with end-organ failure. Unfortunately, the demand for organs far outweighs the supply, leading to long waiting lists and high mortality rates for patients in need [1-3].

The Global Observatory on Donation and Transplantation found that less than 10% of the need for transplants is met annually [4]. Every day, 18 patients die waiting for an organ donation in the United States [5]. The issue is not only because of the low availability of donors but also because of the failure to transfer potential donors to actual donors [6].

Currently, the primary source of organ donation often arises from patients with brain death in the intensive care unit (ICU) or from live organ donors in outpatient settings [7,8]. Because of the shortage of donations, it is crucial to explore other potential sources. One such untapped resource is the emergency department. In the United States, for example, there are 335,000 cardiac arrests annually, of whom 22,000 would be candidates for organ transplantation. When compared to brain death, it is only 10,000 a year [9].

By reviewing the Saudi literature and important organizations, we will see the following: The National Kidney Foundation (NKF) started organ transplantation in Saudi Arabia in 1984. Then, with the progress and expansion of the organs involved in transplantation, such as the liver, lung, and heart, the organization's name changed to SCOT (Saudi Center for Organ Transplantation) in 1993 [10-12]. Organ donation has been encouraged in Islam. It is recited in the Holy Quran and supported by the FATWA of the Council of Senior Scholars (no. 99, dated 6/11/1402 A.H.) [13-14].

Based on the SCOT data, there are 29,000 patients in Saudi Arabia with end-stage renal disease on hemodialysis. The majority are young and middle-aged patients (26-65 years). From 1979 to 2017, only 921 renal transplantations were performed. Living donors account for nearly 84% of all donations. Deceased donations are not common in Saudi Arabia, accounting for less than 15% of all donations. Not surprisingly, 60% of the participants in Bukhari’s study accepted the donation post-death [15].

There are around 194,000 volunteers to donate organs after death in Saudi Arabia in 2021; of them, 69% are between 18 and 30 years of age. In Jabri et al.’s study, 385 Saudis were surveyed, and it was found that 74.1% of them were willing to be organ donors [16]. Unfortunately, most of the time, they are not recognized in the emergency department if they have a cardiac arrest or end-of-life situation [15].

The programs and projects implemented in emergency medicine for organ donation from cardiac-arrested patients have been established in other countries with an acceptable range of success, which led to an increase in the number of donation cases [17]. Fortunately, these days, there are new technical innovations and more competent people, which increases the possibility of success in these circumstances.

The emergency physician's lack of organ donation knowledge and awareness will result in a wasted chance to discover organ donation candidates [18,19]. This is the main goal of this study, which is to determine the degree of awareness and attitudes of emergency physicians toward organ donation in the emergency department. The outcomes of this study will give greater insight into the issue of organ donation and the various paths that organizations interested in organ donation and transplantation may take. To our knowledge, no similar study has been conducted in the literature, addressing the same question and targeting the same type of participants.

## Materials and methods

Study design, setting, and time

This is a cross-sectional study. We include all emergency physicians in adult emergency departments in different centers in Saudi Arabia. We have five main hospitals. There were three hospitals in Riyadh: the Security Forces Hospital, King Khalid University Hospital, and King Faisal Specialist Hospital. One hospital in Taif city was the King Faisal Medical Complex. The last hospital in the Aseer region was Aseer Central Hospital. We selected these hospitals’ physicians so that there would be diversity, including government, tertiary, and educational hospitals. We conducted this survey from July through August 2023.

The questionnaire began with establishing the study's objectives, goals, and target population. Then, we conducted a literature review to identify crucial information that required inquiry and discussion. All the questions have been collected and tailored to cover the main areas. We aimed to have a clear and non-ambiguous question.

The questionnaire content was validated by calculating the content validity ratio for each question. Then, the overall content validity index was calculated, and it was 0.87. The questionnaire was distributed to 10 expert participants to review the survey questions. They have been asked to see any ambiguity among the questions and to ensure relevance and clarity. All their feedback was taken, and the necessary changes have been made. Then, reliability was assessed by repeating the same questionnaire among a specific number of targeted populations, and we found good reliability. After that, the survey was finalized for distribution.

Study participants

We include all emergency physicians working in adult emergency departments across five centers in Saudi Arabia. They should work for at least one year in the emergency department. In addition, they should have a degree in emergency medicine or have been in board training. We exclude pediatric emergency physicians. We categorize all the hospitals into educational, tertiary, and governmental hospital blocks. Then, through computer randomization and selection, we chose the aforementioned five centers. The aim of this randomization is to have different physicians from different hospitals with different experiences. A total of 140 physicians were invited to participate in the study, and we collected 106 responses with a full-answer questionnaire and a response rate of 75.71%.

Data collection

The questionnaire was distributed to all targeted physicians through a link to a Google Form. It included demographics, sex, age, and job title. Furthermore, it included questions about the physician’s knowledge and attitudes about organ donations. In addition, it covered answers to different scenarios they may face in their daily practice regarding organ donation.

Ethical consideration

We got approval from the Security Forces Hospital Institutional Review Board (approval number: 23-676-40). All participants provided consent to participate in the survey.

Statistical analysis

Data analysis was performed using Statistical Package for the Social Sciences (SPSS) Version 23 (IBM Corp., Armonk, NY). Frequency and percentages were used to display categorical variables. Analysis-of-variance test followed by Tukey post-hoc test was used to determine where the exact difference between groups exist. Level of significance was set at 0.05, which is the p-value.

## Results

The sociodemographic profile of the participants is given in Table 1. In terms of age, 76 (71.7%) participants were 20-40 years old, 29 (27.4%) were 41-60 years old, and one (0.9%) was older than 60 years. As for gender, 84 (79.2%) were males, while 22 (20.8%) were females. As for the job title, 19 (17.9%) were service residents, 37 (34.9%) were residents of the Saudi Board of Emergency Medicine, 14 (13.2%) were registrars or equivalents, 10 (9.4%) were senior registrars or equivalents, and 26 (24.5%) were consultants.

**Table 1:** Sociodemographic profile of the participants (N = 106)

Demographical characteristics	N	%
Age		
20-40 years	76	71.70
41-60 years	29	27.40
Older than 60 years	1	0.90
Gender		
Male	84	79.20
Female	22	20.80
Job title		
Service resident	19	17.90
Resident in Saudi board of emergency medicine	37	34.91
Registrar or equivalent	14	13.20
Senior registrar or equivalent	10	9.40
Consultant	26	24.50

**Figure 1:**
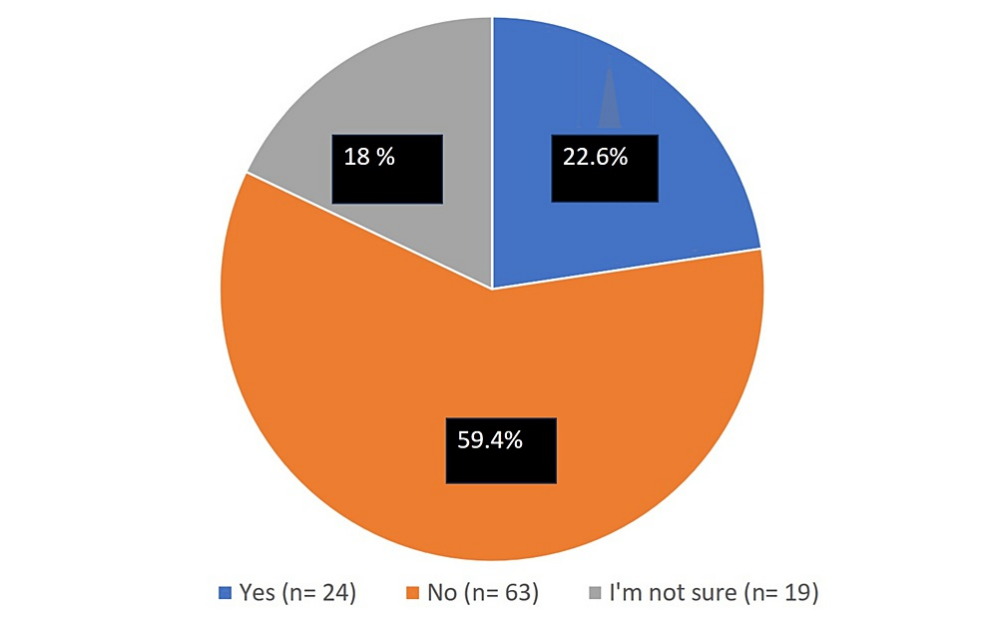
Do you work in a medical center that performs a transplant surgery?

Figure 1 shows the participants’ responses to the question "Do you work in a medical center that performs transplant surgery?". Overall, 24 (22.6%) participants reported working in a medical center that performs transplant surgery, 63 (59.4%) reported that they do not, and 19 (17.9%) reported that they are not sure.

Table 2 presents the emergency physicians’ previous experience with possible organ donation cases and self-assessment toward knowledge regarding their role in organ donation and transplantation. Only 16 (15.1%) physicians recognized a patient as possibly being a candidate for organ donation, 52 (49.1%) doctors reported having very little awareness of the ethical difficulties and conflicts that arise throughout the organ donation procedure, 50 (47.2%) physicians reported having very low knowledge about the SCOT's mission and responsibilities.

**Table 2:** Emergency physicians’ previous experience with possible organ donation case, and self-assessment toward knowledge regarding their role in organ donation and transplantation (N = 106)

Question	N	%
Q1: During your clinical shift, have you ever had to start or report a case as a potential patient or candidate for organ donation?		
Yes, I did	16	15.10
No, I did not	90	84.90
Q2: How would you rate your knowledge of ethical issues and conflicts in the organ donation process?		
Excellent knowledge	5	4.70
Moderate knowledge	43	40.50
Very low knowledge	52	49.10
No knowledge	6	5.70
Q3: How do you rate your knowledge of the Saudi Center for Organ Transplantation's mission and responsibilities?		
Excellent knowledge	4	3.80
Moderate knowledge	32	30.20
Very low knowledge	50	47.20
No knowledge	20	18.80
Q4: At Tawakkalna or the Saudi Center for Organ Transplantation, a large number of people have now signed organ donation forms. Do you have any departmental policies or processes in place that address the early identification of such people as well as how to handle and get in touch with transplantation centers if one of them came to you in a life-threatening situation or had a cardiac arrest?		
Yes, we have	4	3.80
No, we don't have	49	46.20
I don't know	53	50.00

Among the participants, only four (3.8%) reported having departmental policies or processes in place that address early identification for people who signed organ donation forms, as well as how to handle and get in touch with transplantation centers if a donor came into a life-threatening situation or had a cardiac arrest. On the other hand, 49 (46.2%) participants reported that they do not have departmental policies, and 53 (50%) reported that they don’t know.

The results of the questions regarding emergency physicians’ attitudes toward organ donation and transplantation are shown in Table 3. Only seven (6.6%) participants reported attending a workshop or a lecture regarding organ donation and ethical challenges in the emergency department, and 71 (67%) physicians agreed that the emergency department has a role in increasing the number of potential cases of organ donation. Furthermore, 67 (63.2%) physicians reported believing that emergency physicians should be involved in training programs related to organ transplantation and donations.

**Table 3:** Assessment of emergency physicians' attitude toward organ donation and transplantation (N = 106)

Question	N	%
Did you attend any workshop or lecture regarding organ donation and ethical challenges in the emergency department before?		
Yes, I attended (positive answer, 1 score)	7	6.60
No, I did not	99	93.40
Do you agree that emergency department has a role in increasing the number of potential cases of organ donation?		
Yes, I agree	71	67
No, I don't agree	13	12.20
I don't know	22	20.80
Do you believe that the emergency physician should be involved in such training programs related to organ transplantation and donations?		
Yes, I do	67	63.20
No, I don't	20	18.90
I don't know	19	17.90
Do you think that discussing organ donation should be a part of every end-of-life situation in the emergency room?		
Strongly agree	22	20.80
Partially agree	46	43.40
I don't agree	28	26.40
I'm not sure	10	9.40
Establishing an organ donation program in the emergency department will be a necessary step to success in increasing the availability of organ donation cases		
Strongly agree	51	48.10
Partially agree	37	34.90
I don't agree	9	8.50
I'm not sure	9	8.50

Regarding the emergency physician's potential roles in the organ donation process and selection of possible candidates, the results are shown in Table 4. For example, regarding potential patients who could be a possible candidate for organ donation, 49 (46.2%) of the participants voted for patients with a high mortality rate and a poor overall prognosis. Furthermore, 38 (35.8%) physicians chose the DNR (don't resuscitate) cases as an excellent case for organ donation, whereas 35 participants voted for cardiac arrest patients as possible cases for organ donation. On the other hand, 24 (22.6%) physicians said they were not sure, and 11 (10.4%) did not choose any scenario as a potential source.

**Table 4:** Emergency physicians’ potential roles in the organ donation process and selection of possible candidate (N = 106)

Question	N	%
Q1: Which of the following situations, in your opinion, suggests that the patient might be a good candidate for organ donation? (multiple answers can be chosen)		
Patient was brought in while in full cardiac arrest for medical or surgical cases	35	33
The DNR (do not resuscitate) patient	38	35.80
Patients having a high mortality rate who have a bad prognosis, such as, but not limited to, major cerebral bleeding or severe burns	49	46.20
I'm not certain which patients would be suitable	24	22.60
None of the above	11	10.40
Q2: What potential role could an emergency physician play in the process of organ donation? (multiple answers can be chosen)		
Reporting the potential cases for organ donation	65	61.30
Having an active system to recognize the organ donor registered patients right away when they arrive at the emergency department, especially those in life-threatening situations where time is extremely restricted	61	57.50
Increase the teaching and awareness for colleagues and the community	46	43.40
Must have experience and training in organ preservation	33	31.10
Recognizing the ethical considerations and the intense emotions associated with this particular procedure	43	40.60
The fundamental objective should be to prioritize patient survival and improved prognosis, without being influenced by the potential for organ donation	35	33
Join and take part in studies on organ donation.	28	26.40
I'm not sure how to contribute	13	12.30
Q3: Who should explain and interact with the family about the organ donation process and eligibility? (only one answer is possible)		
The emergency physician should be the one	1	0.90
The members of the transplantation and donation teams	64	60.40
The transplantation team and the emergency physician together	32	30.20
I don't know	9	8.50

On the other hand, for the question about possible roles that can be played by the emergency physician in organ donation, most participants (61.5%) indicated reporting to be the most crucial role, whereas 13 (12.3%) participants were not sure how to participate.

In terms of who should initiate the discussion about organ donation with the family, 64 (60.4%) physicians said that it should be the transplantation-trained team, whereas 32 (30.2%) physicians agreed that both the emergency physician and the transplantation team together should approach the family for organ donation initiation.

Table 5 presents the factors associated with emergency physicians’ positive attitude toward their role in organ donation and transplantation. Age, gender, job title, or working in a medical center that performs transplant surgery was not significantly associated with attitude score of emergency physician toward their role in organ donation and transplantation.

**Table 5:** Factors associated with emergency physicians’ positive attitude toward their role in organ donation and transplantation Significant p-value is less than 0.05

Factor	Positive attitude score		P-value
	Mean	Standard deviation	
Age			
20-40 years	3.82	1.73	0.696
41-60 years	3.66	2.24	
Gender			
Male	3.79	1.95	0.817
Female	3.68	1.52	
Job title			
Service resident	3.47	1.58	0.112
Resident in Saudi board of emergency medicine	3.59	1.82	
Registrar or equivalent	3.14	1.92	
Senior registrar or equivalent	5.00	1.33	
Consultant	4.08	2.12	
Do you work in a medical center that performs transplant surgery?			
Yes	4.13	1.75	0.420
No	3.57	1.91	
I'm not sure	3.95	1.87	

## Discussion

This study demonstrates that the majority of the participants did not initiate or engage in discussions regarding organ donation during their clinical duties. In addition, more than 50% of candidates have limited knowledge of the organ donation process and SCOT missions and services. Moreover, more than half of the participants agreed that the emergency physician has to learn more about organ donation ethics, policy, and procedure. They concurred that they can play multiple roles in increasing the number of organ donation cases. Most of the participating physicians agreed that we should discuss organ donation in each end-of-life case in the emergency department.

Most candidates agreed that the organ donation and transplantation teams should be the ones to contact the family regarding organ donation. This agreement was comparable to the Macvean Michael et al.'s studies [9,13]. The reason for this is that most emergency physicians prefer not to have a conflict of interest. The legality is to concentrate on patient survival and to offer all essential assistance to preserve the patient's life, which is always the first priority.

Macvean et al.'s research, like ours, demonstrates that emergency physicians' lack of awareness is clear and is connected with a lack of opportunity to discover organ donation instances. Nevertheless, the emergency physicians in our study state that they could be able to play different roles in organ donations. These possible roles include, but are not limited to, reporting the potential cases of organ donation, contributing to related research, and being aware of all difficulties and ethical issues in organ donation in the emergency department. Among all roles, the most critical role of emergency medicine in organ donation is early and prompt notification of candidate potential cases for organ donation with a focus on full resuscitation. These findings support what has been concluded in Iserson et al.’s study [16]. This signifies that the emergency physician is willing to engage in the organ donation process.

In Kim et al.’s study [17], implementing a program in the emergency department improved the effectiveness of recruiting more organ donation candidates. It corroborates the findings of our study. These results support our hypothesis. It states that a lack of knowledge and well-established programs in the emergency department will result in lost potential cases and organ donation opportunities.

Different results were found in Kondori et al.’s study [20]. It concludes that there is a high level of knowledge among emergency physicians and nurses toward organ donation in the emergency department. This result differed from ours because their study was conducted exclusively at academic, educational, and teaching hospitals affiliated with a variety of institutions and universities. In our study, we included different hospitals. It consisted of academic, tertiary, and government hospitals. Therefore, we included physicians from diverse hospital backgrounds and facilities.

Haddiya et al. conducted a survey of medical students, lawyers, and nurses in Morocco. It was a cross-sectional study. They found that there was a low and inadequate degree of awareness, which is consistent with the findings of our research [21]. Furthermore, in a Hungarian research conducted by Kanyari et al., the similar lack of awareness across different health practitioners was reported [22]. In addition, Rios et al. conducted a cross-sectional study in Spanish and concluded that there is a lack of knowledge among the medical students [23]. Chandrasekaran et al.'s study, which also highlights the knowledge gap and improvement needs among Indian healthcare providers, supports the findings of our study as well [24]. All previous studies have shown that there is a great demand for education among healthcare providers.

We can attribute the lack of knowledge and attitudes to the lack of education as well as the unavailability of the policies and procedures in the emergency departments. In addition to the complexity of the situation, the medical and ethical challenges overlapped at the same time. To overcome such a challenge, there must be clear policies and procedures in the emergency departments.

This study has multiple limitations. First, there was no valid tool or measurement to assess the knowledge and attitudes of the physician in the literature. For that reason, we created a new survey with the appropriate validation measures. Secondly, the recall and non-responder bias are general limitations in such types of studies. We tried to limit the number of recall events questions, and we limited the number of non-responders by following them days later to reply and participate. Thirdly, the unique viewpoint of the administrative personnel was not sought. Finally, there were no pediatric emergency physician involvements. It may be an area of research in the future.

The exclusion of pediatric emergency physicians was for the purpose of specifying the targeted population. It does not affect the results because most of the cardiac deaths in hospitals or outside hospitals occur in the adult population more than in the pediatric population. The other reason is that volunteer organ donations are only in the adult population. Regarding the recall bias, we aim to limit the questions related to it, and if there is a question that requires remembering a major event such as organ donation, it is not easy to forget and should not affect the results.

## Conclusions

There are so many patients with end-organ failure nowadays. Unfortunately, their numbers increase every day. The only definite intervention to save their lives so far is organ donation. Unfortunately, the number of available donors is much less than what is needed. So, looking for sources for organ donation other than brain-dead donors in the ICU or living donors is a rational thought. The emergency department is a valuable resource, and it should be utilized. There are so many critical cases and end-of-life cases in the emergency department. In our study, we conclude that there is an apparent lack of knowledge and rarely implemented policies and procedures in the emergency departments. The recommendation is to initiate such a program and start educating the physicians to improve the organ donation pools in our healthcare facilities.

We hope that the study’s findings and publication will raise awareness of this possibility in the emergency department among all societies engaged in organ donation and transplantation. This should result in increased educational programs and the implementation of the required policies and procedures.

## Appendices

Organ Donation Questionnaire

1. How old are you?

·        20-40

·        41-60

·        >60

2. Gender

·        Male

·        Female

3. Job title

·        Resident service

·        Resident in Saudi board of emergency medicine

·        Registrar or equivalent

·        Senior registrar or equivalent

·        Consultant

4. Do you work in a medical center that performs transplant surgery?

·        Yes

·        No

·        I’m not sure

5. Have you registered as an organ donor with Tawakkalna or the Saudi Center for Organ Transplantation (SCOT)?

·        Yes

·        No

·        Possibly in the future

6. How would you rate your knowledge of ethical issues and conflicts in the organ donation process?

·        Excellent knowledge

·        Moderate knowledge

·        Very low knowledge

·        No knowledge

7. How do you rate your knowledge of the Saudi Center for Organ Transplantation’s mission and responsibilities?

·        Excellent knowledge

·        Moderate knowledge

·        Very low knowledge

·         No knowledge

8. Did you attend any session or lecture regarding organ donation opportunities and ethical challenges in the emergency department before?

·        Yes, I did

·        No, I did not

9. Do you agree that the emergency physician has a role in increasing the number of potential cases of organ donation?

·        Yes, I agree

·        No, I don’t agree

·        I don’t know

10. Do you believe that the emergency physician should be involved in such training programs related to organ transplantation and donations?

·        Yes, I do

·        No, I don’t

·        I’m not sure

11. Who should explain and interact with the family about the organ donation process and eligibility?

·        I believe the emergency physician should be the one

·        I believe members of the transplantation and donation teams should communicate with them

·        I believe that the transplantation team and the emergency physician should work together

·        I don’t know

12. During your clinical shift, have you ever had to start or report a case as a potential patient or candidate for organ donation?

·        Yes, I did

·        No, I didn’t

13. At Tawakkalna or the Saudi Center for Organ Transplantation, a large number of people have now signed organ donation forms. Do you have any departmental policies or processes in place that address the early identification of such people as well as how to handle and get in touch with transplantation centers if one of them came to you in a life-threatening situation or had a cardiac arrest?

·        Yes, we have

·        No, we don’t have

·        I don’t know

14. Which of the following do you believe is the best scenario for organ donation in your emergency department? (you may select multiple answers)

·        Patient was brought in while in full cardiac arrest for medical or surgical cases

·        Cardiac arrest occurring within a few hours or days of arrival at the emergency department

·        The DNR (do not resuscitate) patient

·        Patients having a high fatality rate who have a bad prognosis, such as, but not limited to, major cerebral bleeding or severe burns

·        I’m not certain which patients would be suitable

·        None of the above

15. What potential role could an emergency physician play in the process of organ donation? (you may select multiple answers)

·        Reporting possible organ donation cases

·        Having an active system to recognize the organ donor registered patients right away when they arrive at the emergency department, especially those in life-threatening situations where time is extremely restricted

·        Increase the teaching and awareness for colleagues and the community

·        Must have experience and training in organ preservation

·        The recognition of the ethical considerations and the intense emotions associated with this particular procedure

·        The fundamental objective should be to prioritize patient survival and improved prognosis, without being influenced by the potential for organ donation

·        Join and take part in studies on organ donation

·        I am uncertain of how to contribute

16. Do you believe that every end-of-life case in the emergency room should include a discussion regarding organ donation?

·        Strongly agree

·        Partially agree

·        Don’t agree

·        I’m not sure

17. In order to successfully increase the number of organ donation cases, the emergency department must establish a program for organ donation.

·        Strongly agree

·        Partially agree

·        Don’t agree

o   I’m not sure
